# Mental status is significantly associated with low back pain: a survey-based cross-sectional study among Japanese women

**DOI:** 10.1186/s13104-023-06276-4

**Published:** 2023-01-30

**Authors:** Mayumi Watanabe, Chikako Tomiyama, Takuya Nikaido, Tokimasa Takeda, Nozomu Mandai

**Affiliations:** 1 Faculty of Health Sciences, Kansai University of Health Sciences, Osaka, Japan; 2 Faculty of Science and Engineering, Chuo University, Tokyo, Japan; 3 Graduate School of Health Sciences, Niigata University, Niigata, Japan; 4 Department of Orthopaedic Surgery, Fukushima Medical University School of Medicine, Fukushima, Japan; 5 Zinbunken, Kyoto University, Kyoto, Japan; 6 Faculty of Nursing Science, Tsuruga Nursing University, Tsuruga, Japan; 7 Ibaraki Prefectural University of Health Sciences, Ibaraki, Japan

**Keywords:** Low back pain, Body temperature, *Hie*, Stress

## Abstract

**Objective:**

Low back pain (LBP) is a highly prevalent condition that poses significant patient burden. This cross-sectional study identified factors associated with LBP occurrence and developed a strategy to identify, prevent, and reduce LBP-related burden on patient health. A web-based questionnaire-answering system was used to assess the potential effects of LBP on mental health, assessing five domains (physical features, demographics, lifestyle, diet, and mental status) conceptually associated with *hie*, a common disease state traditionally described in the Japanese culture as a chilly sensation.

**Results:**

Of 1000 women, 354 had and 646 did not have LBP. The Chi test identified 21 factors, and subsequent multivariate logistic regression indicated eight factors significantly associated with LBP: age, history of physician consultation regarding anemia, history of analgesic agents, dietary limitations, nocturia, sauna use, *hie*, and fatigue. Furthermore, women with LBP exhibited a significantly lower body temperature (BT) in the axilla/on the forehead than women without LBP. LBP and *hie* are subjective and potentially affected by patient mental status. Stress reduces blood circulation, causing hypothermia and possibly worsening LBP. Therefore, mental-health support is important for patients with LBP to reduce physiological stress. Hyperthermia therapy, a traditionally prescribed intervention, is a potential intervention for future studies.

**Supplementary Information:**

The online version contains supplementary material available at 10.1186/s13104-023-06276-4.

## Introduction

Many low back pain (LBP) patients show substantial discrepancies between the objective observations of medical practitioners and their subjective complaints, suggesting causes other than physiological disorders [1]. Particularly, 85% of chronic LBP (CLBP) cases are nonspecific, with no identified physiological, neural, or orthopedic spine disorders [2]. LBP frequently causes absenteeism among healthcare workers, nurses, and factory workers and profoundly affects the patients, society, and economy [3]. Consequently, LBP prevention requires a greater understanding of the causative factors to reduce the likelihood of its onset. Early detection/treatment should be the primary concern to achieve this goal. Psychological effect or social stress significantly affects LBP [4].

A recent study hypothesized that acute LBP might develop into CLBP (LBP lasting > 3 months) in the presence of psychological stress or other mental/emotional factors [1]. In Japan, LBP is a common ailment. The Japanese Health, Labour and Welfare Ministry regularly reported that the ratio of LBP prevalence/consultation is significantly high consistently [5]. The Japanese Orthopaedic Association recently developed a pain evaluation questionnaire assessing five key domains of LBP, including mental health [6–8]. It includes questions regarding physical and mental health, and many doctors focus on the mental health of LBP patients. This questionnaire was used to improve LBP identification and support targeted therapeutic strategies for efficient, multimodal interventions; however, LBP prevalence has not decreased, despite the questionnaire’s implementation.

Therefore, for this study, we prepared a new questionnaire comprising five domains (physical features, demographics, lifestyle, diet, and mental status) and further investigated the effects of mental status on LBP using body temperature (BT). Additionally, we utilized an original parameter, “*hie,*” a chilly or cold sensation. In Japan, *hie* is used to describe the subjective, uncomfortable feeling of coldness; females are 2.7 times more likely to suffer from it than males [5]. Although this term is very common in Japan, it has not been conceptually well-established in modern medicine owing to cultural and language barriers. We conducted a cross-sectional study on various factors using a questionnaire to investigate the background and cause of LBP, assess the possible effects of mental status on LBP, and propose a new approach to prevent LBP, particularly nonspecific LBP. We sought a new strategy to identify, prevent, and reduce the LBP-related health burden while considering early detection, BT assessment, traditional medicinal concepts, and effects of LBP on patient mental health.

## Main text

### Methods

#### Participants

Participants were recruited from a database maintained by a Japanese survey company (Cross Marketing Inc., Tokyo, Japan). The questionnaires were administered using an internet-based survey conducted by this company. Among 300,000 individuals listed in the database, 5000 were selected using random sampling, stratified by age and place of residence. This subsample was resampled by proportional allocation to balance the sex ratio, yielding a final sample size of 1000 individuals who were recruited. All participants were asked to use the web-based questionnaire-answering system. All participants who accessed the survey web page provided written informed consent before starting the questionnaire, and all of them also responded to the questionnaire. Owing to the BT recording location criteria, women without access to a contact-free thermometer (infrared thermometer) were excluded.

#### Low back pain

LBP refers to pain, stiffness, decreased lower back movement, and difficulty in straightening one’s lower back. Participants with or without LBP were divided into two groups: LBP ( +) (n = 354, 35.4%) and LBP ( −) (n = 646, 64.6%). Our questionnaire (Additional file 1) recorded clinical details, including doctor visits or diagnostic imaging. Participants were asked to report on various aspects of their LBP condition [6–8].

#### Hie

Hitherto the “*hie*” parameter has been uninvestigated; therefore, we incorporated it with regard to LBP into our new questionnaire. In traditional medicine, *hie* is known to induce pain, including in LBP patients [9]. Local or small-scale studies have been conducted; however, these cannot be considered sufficient evidence to explore the effect of *hie* on LBP [10–12]. We quantified *hie* (a traditional concept) by measuring BT (a modern concept) because, according to recently published studies, *hie* may be influenced by multiple factors, including mental status [13–16]. Other information that may affect both BT and *hie*, including current place of residence, place of birth, room temperature (RT) while answering the questionnaire, current dietary consumption, and mental status, was obtained through the questionnaire.

##### (i) Physical features

This domain included the following parameters: age, breathing rate, and body mass index (BMI). Participants were asked to select the appropriate age category. Simultaneously, participants were asked to measure and select their breathing rate.

As in previous studies, we calculated the BMI [17–19] by inquiring about the participant’s weight and height and applying the following formula:

$${\text{BMI}}\, = \,{\text{weight }}\left( {{\text{kg}}} \right)\, \div \,{\text{height }}\left( {\text{m}} \right)^{{2}}$$

Participants were categorized into three groups based on their BMI (< 18.5, 18.5–24.9, and ≥ 25.0).

##### (ii) Demographics

This domain included the following parameters: present residence, place of birth, and occupation. Social situations may influence LBP; therefore, we asked the participants to provide information about their current place of residence (present residence) and place of birth. We also inquired about their occupation.

##### (iii) Lifestyle factors

Living conditions, including RT [20–22], presence of a room heater and usage time, winter wear use, and air-conditioner use, might affect LBP. We asked participants to provide their RTs, history of heater use, usage time duration, use of winter wear, likability of the air-conditioner, history of consultations with a doctor regarding anemia, and history of analgesics use. Data on dietary limitations, menstrual history, recent history of catching a cold, and smoking history were also collected [17, 23].

Additionally, we asked the participants whether or not they exercised [24], the type of exercise performed, duration of time spent sitting in one position, duration of sleep [25–27], bedtime, frequency of going to the bathroom during sleeping hours (nocturia), bathing habits, and history of sauna use [28–30].

“Mild *hie*” is the sensation of feeling chilly, whereas “severe *hie*” indicates feelings of discomfort [10–13]. Therefore, we asked the participants regarding the presence or absence of *hie*.

##### (iv) Diet

According to previous studies, we asked the participants to rate their food intake frequency [31], especially cold foods, including fish, beans, fermented food, and richly flavored foods, as well as their taste and distaste for food.

##### (v) Mental status

Although the causes of LBP are difficult to identify, researchers have reported the possible influence of mental status on LBP [4, 32]. Anger may exacerbate LBP [33–36]; therefore, participants were asked to report their emotional experience [24].

#### Statistical analysis

All statistical analyses were performed using IBM SPSS Statistics software for Windows, version 25.0 (IBM Corp., Armonk, NY, USA). Means (± standard deviations) were used to characterize the distributions of continuous variables.

First, Pearson’s chi-squared test was performed to study both groups (LBP [ −] or [ +]) and determine candidate factors for the multivariate analysis. Next, multiple logistic regression analysis with forward stepwise model selection was performed to determine the factors significantly associated with LBP. The values are presented as 95% confidence interval and adjusted odds ratio (OR). The dependent variable in the multiple logistic regression models was binary: LBP ( −) and LBP ( +), and the null hypothesis was that the probability of observing a regression coefficient of 0 or 1 is influenced solely by chance rather than by any of the independent variables. We also conducted Student’s t-test and analysis of variance to study LBP.

All statistical tests were two-tailed, and a statistical significance was set at P < 0.05.

## Results

Table 1 shows the participant’s basic characteristics. Among 1000 participants, 354 (35.4%) were grouped as LBP ( +) participants. Pearson’s chi-square test identified 21 factors in the five domains that were significantly associated with LBP. Many factors in the mental status domain showed a highly significant association with LBP.

**Table 1:** Characteristics of the LBP ( −) and LBP ( +) groups

Characteristics	LBP ( −)		LBP ( +)		P
	n	%	n	%	
Total (n = 1000)	646	64.6	354	35.4	
*Physical features*					
**Age (years)**					0.001**
20–29	145	73.2	53	26.8	
30–39	174	69.3	77	30.7	
40–49	180	60.8	116	39.2	
50–59	147	57.6	108	42.4	
*Lifestyle*					
**Breathing rate (/min)**					0.047*
< 15	292	68.1	137	31.9	
≥15	354	62.0	217	38.0	
**Duration of heater use**					0.005**
Whole sleeping duration	68	56.2	53	43.8	
Until waking up	35	53.0	31	47.0	
Until falling asleep	46	74.2	16	25.8	
Before waking up & until falling asleep	25	52.1	23	47.9	
Do not use heater while sleeping	439	67.7	209	32.3	
Do not use heater at all	33	60.0	22	40.0	
**History of consultations with a doctor regarding anemia**					< 0.001***
Currently undergoing anemia treatment	13	48.1	14	51.9	
History of anemia treatment	50	52.1	46	47.9	
Anemia present but not undergoing treatment	64	50.8	62	49.2	
Presence of diseases other than anemia	57	60.6	37	39.4	
Did not consult a doctor	462	70.3	195	29.7	
**History of analgesics use**					< 0.001***
2/week	20	51.3	19	48.7	
1/week	28	45.2	34	54.8	
3/month	65	57.0	49	43.0	
Sometimes	140	55.3	113	44.7	
No history of use	393	73.9	139	26.1	
**Dietary limitations**					0.001**
Ongoing	145	60.4	95	39.6	
Sometimes	300	61.5	188	38.5	
No limitations	201	73.9	71	26.1	
**Menstrual history**					< 0.001***
Irregular	115	55.0	94	45.0	
Painful	155	62.5	93	37.5	
No problem	286	71.9	112	28.1	
Absence of menstruation	90	62.1	55	37.9	
**Recent history of catching a cold**					0.028*
No history of a cold	515	66.8	256	33.2	
Last October ~ no (~ September yes)	83	57.6	61	42.4	
Last October ~ yes	48	56.5	37	43.5	
**Smoking history**					0.009**
No history	501	66.5	252	33.5	
Sometimes	18	42.9	24	57.1	
Very often	64	58.7	45	41.3	
Smoked/not now	63	65.6	33	34.4	
**Duration of sleep (hours)**					0.013*
< 6	222	59.0	154	41.0	
6–7	214	66.7	107	33.3	
≥7	210	69.3	93	30.7	
**Frequency of using the bathroom at night (nocturia)**					< 0.001***
0	414	70.5	173	29.5	
1	181	57.3	135	42.7	
2	34	52.3	31	47.7	
≥3	17	53.1	15	46.9	
**History of sauna use**					< 0.001***
Yes	31	40.3	46	59.7	
No	615	66.6	308	33.4	
***Hie***					0.007**
Yes	328	68.9	148	31.1	
No	318	60.7	206	39.3	
*Diet*					
**Tastes and distastes**					0.001**
Yes	88	51.8	82	48.2	
N/A	244	67.6	117	32.4	
No	314	67.0	155	33.0	
**Cold foods**					0.004**
Frequently	265	59.4	181	40.6	
N/A	202	71.1	82	28.9	
Hardly	179	66.3	91	33.7	
**Consumption of richly flavored foods**					0.020*
Frequently	189	58.5	134	41.5	
N/A	272	67.2	133	32.8	
Hardly	185	68.0	87	32.0	
*Mental status*					
**Feelings of anger**					0.013**
Yes	152	58.5	108	41.5	
N/A	348	64.9	188	35.1	
No	146	71.6	58	28.4	
**Feelings of inferiority**					0.004**
Yes	265	70.1	113	29.9	
N/A	251	63.9	142	36.1	
No	130	56.8	99	43.2	
**Feelings of deteriorating health**					0.001**
Yes	109	56.2	85	43.8	
N/A	261	62.4	157	37.6	
No	276	71.1	112	28.9	
**Feelings of exhaustion**					< 0.001***
Yes	153	56.5	118	43.5	
N/A	239	62.1	146	37.9	
No	254	73.8	90	26.2	
**Feelings of failure**					< 0.001***
Yes	223	57.8	163	42.2	
N/A	196	63.4	113	36.6	
No	227	74.4	78	25.6	

*LBP* low back pain; *BMI* body mass index; *RT* room temperature

^*^P < 0.05

^**^P < 0.01

^***^P < 0.001

The proportion of LBP ( +) cases increased from 6.8% in the “20–29” age group to 30.7% in the “30–39” age group, 39.2% in the “40–49” age group, and 42.4% in the “50–59” age group. Ninety-nine LBP ( +) participants consulted doctors, while 255 did not. Of these 354 participants, 36 had specific LBP with a physiological cause; the remaining participants (318) had nonspecific LBP with an undetermined physiological cause.

The multivariate logistic regression analysis identified eight factors associated with LBP (Table 2). Significant differences were observed between the age groups “20–29” and “40–49” and between the “20–29” and “50–59” by analysis of variance/post hoc analysis. No significant difference was found between the four groups with respect to height and weight.

**Table 2:** Results of multivariate logistic regression analysis

Parameters	Co-efficient	SE	P	OR	95% CI	
					Lower	Upper
**Age (years)**						
20–29	–	–	–	1.000		
30–39	0.901	0.230	0.000***	0.406	0.259	0.637
40–49	0.776	0.206	0.000***	0.460	0.307	0.689
50–59	0.340	0.189	0.072	0.712	0.491	1.031
**History of consultations with a doctor regarding anemia**						
Currently undergoing anemia treatment	–	–	–	1.000		
History of anemia treatment	0.756	0.430	0.079	2.129	0.916	4.951
Anemia present (No treatment)	0.614	0.240	0.010*	1.848	1.155	2.954
Presence of diseases other than anemia	0.725	0.212	0.001**	2.064	1.362	3.130
Did not consult a doctor	0.377	0.245	0.124	1.458	0.902	2.355
**History of analgesics use**						
2/week	–	–	–	1.000		
1/week	0.583	0.361	0.106	1.792	0.883	3.639
3/month	1.078	0.301	0.000***	2.939	1.628	5.304
Sometimes	0.732	0.231	0.002**	2.080	1.323	3.269
No history	0.751	0.171	0.000***	2.119	1.516	2.961
**Dietary limitations**						
Ongoing	–	–	–	1.000		
Sometimes	0.491	0.210	0.019*	1.634	1.083	2.465
No limitations	0.384	0.182	0.035*	1.468	1.028	2.096
**Frequency of using the bathroom at night (nocturia)**						
0	–	–	–	1.000		
1	0.527	0.412	0.201	0.590	0.263	1.324
2	0.016	0.419	0.969	0.984	0.433	2.237
≥3	0.092	0.484	0.849	0.912	0.353	2.357
**Sauna use**						
Yes	–	–	–	1.000		
No	1.049	0.276	0.000***	2.854	1.662	4.900
**Hie**						
No	–	–	–	1.000		
Yes	0.183	0.070	0.009**	1.201	1.047	1.378
**Feeling exhaustion**						
Yes	–	–	–	1.000		
N/A	0.473	0.174	0.007**	0.623	0.443	0.876
No	0.126	0.178	0.480	1.134	0.800	1.609
**Constant**	1.025	0.479	0.032*	0.359		

*CI* confidence interval; *OR* odds ratio; *SE* standard error

^*^P < 0.05

^**^P < 0.01

^***^P < 0.001

The reasons for using these drugs include: “during menstruation” (386 [223 + 163] participants) and “back/knee pain” (46 [12 + 34] participants; 73.9% of them were LBP [ +]).

The main way of obtaining these drugs is via the “drug store/internet” rather than through a “doctor consultation.”

## Discussion

Our findings identified eight factors that are significantly associated with LBP: age, history of consultation with a doctor regarding anemia, history of analgesics use, dietary limitations, nocturia, sauna use, *hie*, and feeling exhausted. We also found that participants with LBP had a significantly lower BT in the axilla/on the forehead than participants without LBP. These findings suggest that stress is the root cause of LBP owing to its association, both physically and mentally, with the eight identified factors. These findings shed light on the substantial discrepancies between objective observations of medical practitioners and subjective patient accounts of LBP.

**Fig. 1:**
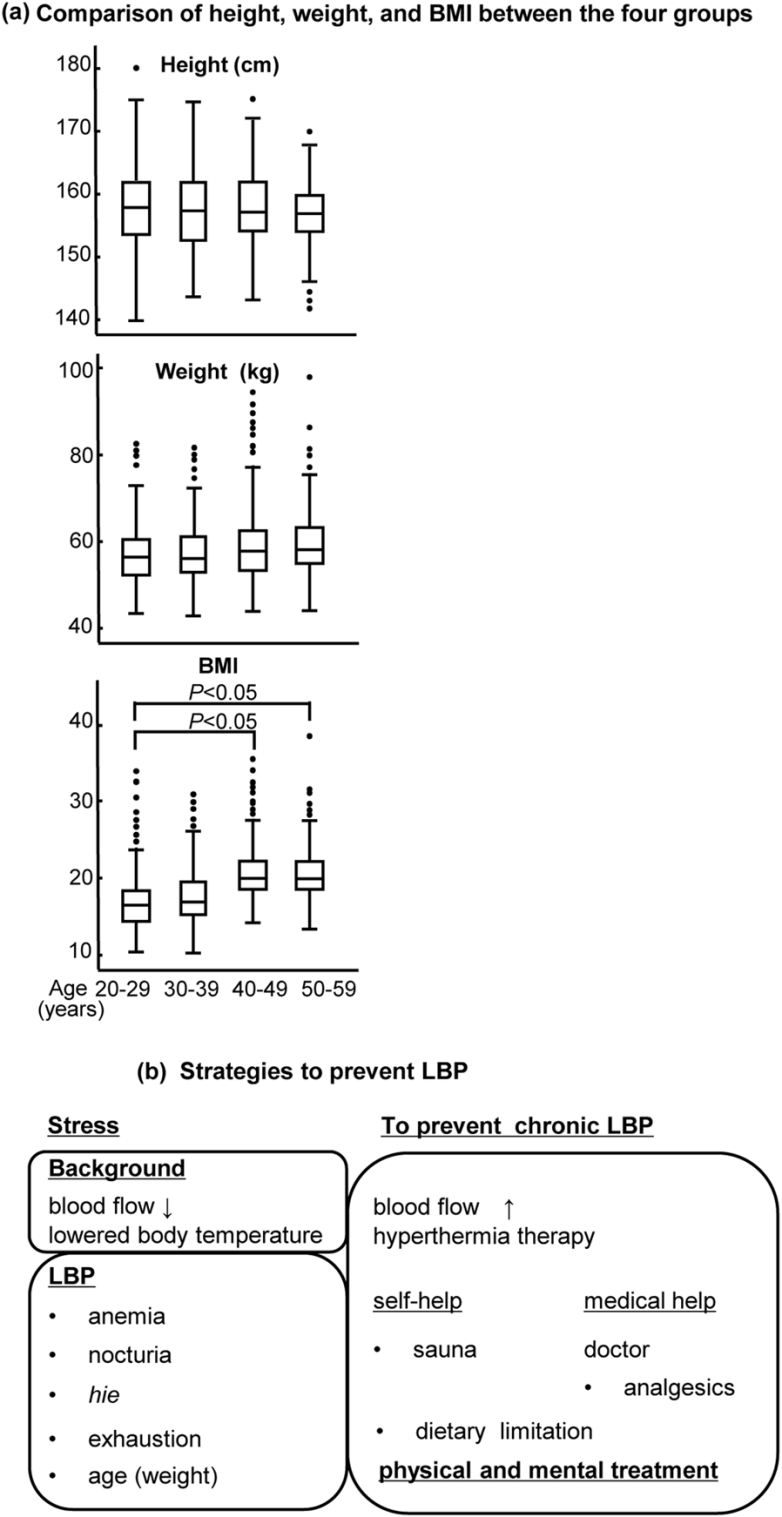
**a**Comparison of height, weight, and BMI between the four groups. **b** Strategies to prevent LBP. LBP, low back pain; BMI, body mass index

Lumbar spine degeneration or muscular weakness may influence LBP. However, our results demonstrated that increase in weight and BMI with age were significantly associated with LBP (Fig. 1a) and may play a role in inducing LBP. The increase in weight/BMI with age may be why age was identified as a factor by multivariate logistic regression analysis (Table 2). We initially postulated that anemia might play a role in influencing LBP; however, high ORs for “history of anemia, no treatment” and “diseases other than anemia” may imply that many LBP patients who consult doctors do not suffer from “anemia.” Rather, LBP is the reason for visiting the doctor.

Analgesics are designed for anti-inflammatory action and pain relief. To understand the reason ORs for “3(/month),” “sometimes,” and “no history” were higher than those for “2/(week),” we conducted further analysis of the 468 participants with a history of use of these drugs. The reasons and manner of obtaining analgesics (Tables 3 and Table 4) could explain the reason why the ORs for “3/month” and “sometimes,” which allude to menstruation, were higher than those for “2/week.” Therefore, it appears that participants used the drugs during menstruation and without obtaining a doctor’s prescription. A *p* value of < 0.001 for “menstruation” (Table 1) supports this understanding.

**Table 3:** Reasons for analgesics use (n = 468)

	LBP (−)		LBP ( +)	
	n	%	n	%
During menstruation	223	57.8	163	42.2
Back/knee pain	12	26.1	34	73.9
Skin disorder/fever	18	50.0	18	50.0

*LBP* low back pain

**Table 4:** Manner of obtaining analgesics (n = 468)

	LBP (−)		LBP ( +)	
	n	%	n	%
After consultation with a doctor (Doctor’s prescription)	47	45.2	57	54.8
Drug store/internet	187	56.2	146	43.8
Friend/family	19	61.3	12	38.7

*LBP* low back pain

Regarding “dietary limitations,” it is known that many Japanese women are on restricted diets [37]. However, the percentage of LBP (−) participants with “no dietary limitations” is 73.9% (Table 1). This may imply that LBP (−) participants do not have to be on dietary limitations as much as LBP ( +) participants do because they do not have to lose weight to manage LBP. In this study, the weight of LBP ( +) participants was significantly higher than that of LBP (−) participants (Table 5).

**Table 5:** Comparison of characteristics between LBP ( −) and LBP ( +) participants

Characteristics	LBP (−)	LBP ( +)	P
Height (cm)	157.74 ± 5.51	158.55 ± 5.68	0.638
Weight (kg)	51.70 ± 8.46	54.11 ± 10.39	0.002**
BT, axilla (℃)	36.18 ± 0.36	36.13 ± 0.42	< 0.000***
BT, forehead (℃)	36.11 ± 0.35	36.03 ± 0.39	0.003**
BT, hand (℃)	34.33 ± 0.54	34.31 ± 0.57	0.179
BT, foot (℃)	32.23 ± 0.56	32.28 ± 0.57	0.456
Highest recorded BT (℃)	36.22 ± 0.34	36.18 ± 0.41	< 0.001***
Lowest recorded BT (℃)	32.23 ± 0.56	32.28 ± 0.57	0.456
Maximum BT difference (℃)	3.99 ± 0.59	3.90 ± 0.59	0.543
BMI	20.72 ± 3.16	21.46 ± 3.89	0.001**

LBP ( +) participants had a significantly lower BT in the axilla and on the forehead than LBP (−) participants

*LBP* low back pain; *BT* body temperature; *BMI* body mass index

^*^P < 0.05

^**^P < 0.01

^***^P < 0.001

The percentage of LBP ( +) participants who frequented the bathroom at night increased; 29.5%, 42.7%, 47.7%, and 46.9% of participants frequented the bathroom 0, 1, 2, and ≥ 3 times, respectively. This result may be useful to study LBP and to prevent accidental orthopedic conditions, including bone fracture or leg sprain. BT (axilla) of LBP ( +) participants was lower than that of LBP ( −) participants (Table 5), which may increase the frequency of night bathroom visits, although this needs further study. Bathroom visits at night are considered dangerous as they increase the risk of falls, which may result in injury in LBP ( +) participants.

In our study, only 77 participants indicated “sauna” use; however, interestingly, 59.7% of them were LBP ( +). LBP ( +) participants might experientially know that hyperthermia stimulation can ease LBP. Table 5 implies that the BT in the axilla/on the forehead reflects the BT in the trunk. The BT in the axilla is the deep (inside) BT and that of the forehead is the surface BT. Other BTs are away from the center (peripheral). This result may explain that LBP ( +) participants had lower BTs and may need hyperthermia stimulation.

Traditional medicine often uses hyperthermia stimulation; moxibustion stimulation eases LBP [38, 39], and so does hot spring therapy [40, 41]. The Japanese Orthopaedic Association conducted a systematic review analysis in 2022 and reported the efficiency of hyperthermia therapy for LBP management [42]. In Japan, many women suffer from *hie*, and this concept is not quite popular outside Japan. Although the study of *hie* alone is insufficient, both *hie* and LBP are understood subjectively. A recent study revealed a strong relationship between these two sensations [1].

As discussed above, LBP ( +) participants had a significantly lower BT in the axilla/on the forehead than that of LBP ( −) participants. This may be attributed to the fact that LBP ( +) participants are not as physically active as LBP (−) participants. Pain may weaken muscular action and muscle pumping, which circulates warm blood from the heart to the axilla.

Both *hie* and LBP are felt subjectively, and thus, they can be easily affected by mental status. Table 1 showed *p* values of < 0.05 for five of seven questions in the mental status domain. Interestingly, the multivariate logistic regression analysis identified only “feeling exhausted” as a factor (Table 2), and Table 1 shows a decrease in the percentage of LBP ( +) participants with the factor of “feeling exhausted” as follows: “yes” (43.5%), “N/A” (37.9%), and “no” (26.2%). It implies a strong association between the factor of “feeling exhausted” and LBP. Many researchers reported the effect of mental status on LBP. LBP ( +) participants may feel exhausted in daily life because of pain.

Increases in body weight and BMI affect LBP. Researchers reported the effect of mental status on factors associated with LBP, especially in CLBP [1]. In Table 1, the breathing rate (/min) of LBP ( +) participants was “ < 15” for 31.9% and “ ≥ 15” for 38.0%. Stress can increase breathing rate via the autonomic nervous system [43, 44]. Moreover, the result of the mental status domain in Table 2 shows the emotional status of the participants; stress manifested by emotions, including “anger,” “feeling of inferiority,” “deteriorating health,” “feeling exhausted,” and “feeling unsuccessful,” rather than “feeling happy.” Stress induces adrenaline stimulation, reduces blood circulation, and causes hypothermia under sympathetic nerve dominance [45, 46]. It may increase the frequency of night bathroom visits. Decreased blood circulation or lowered BT may worsen LBP. To avoid pain, some LBP ( +) patients consult a doctor while some adopt dietary limitations to reduce weight.

LBP **( +)** patients often show substantial discrepancies between the objective observations of medical practitioners and patients’ subjective complaints. It is well known that stress affects mental status, and thus, researchers began to investigate mental status/attitude [47]. Currently, many workplaces regularly evaluate employees’ stress levels. As LBP ( +) participants demonstrated the factor of “feeling exhausted” in the mental status domain, we need to pay heed to their complaints and take effective measures.

Medical staff plays an important role in assisting LBP ( +) patients to prevent back pain from developing into a chronic/more severe pain. For example, fall-prevention strategy during nighttime bathroom visits and purchase of may help. Abuse of analgesics, which can be obtained over the counter or via the internet, may affect blood flow. Analgesics are inhibitors of cyclooxygenase, which stops prostaglandins production, and whole-body blood circulation is inhibited [48]. Therefore, LBP mechanism and appropriate drug usage should be properly understood (Fig. 1b).

*Hie* has not been researched thoroughly to date; however, both *hie* and LBP are undesirable sensations. Traditional medicine considers *hie* a harbinger of various diseases, and a common traditional prescription is hyperthermia therapy (sauna or hot spring). A recent study revealed strong effects between *hie* and LBP [1], and thus it is also important to investigate traditional therapy for LBP reduction.

## Conclusions

Stressful lifestyles are a common part of modern society and may be a strong risk factor for LBP development, especially considering poor mental health status, decreased BT, and poor blood circulation. LBP is a physical disease; however, it may also involve mental health factors. We, therefore, recommend that timely diagnosis and treatment of psychological stressors and mental health counseling could help minimize LBP incidence.

## Limitations

First, the sample size was small. Second, only participants who frequently use the internet could participate. Therefore, our results may not be representative of the wider population. Nonetheless, these factors may be valuable to assess in future studies, and some conclusions drawn here will lead to further debate.

## Supplementary Information

**Additional file1.** Questionnaire.

## Data Availability

The datasets used and/or analyzed during the current study are available from the corresponding author on reasonable request.

## Abbreviations

LBP: Low back pain
CLBP: Chronic low back pain
BT: Body temperature
BMI: Body mass index
RT: Room temperature
