# Supplementary material for: Mental status is significantly associated with low back pain: a survey-based cross-sectional study among Japanese women
Source: BMC Res Notes. 2023 Jan 30;16:8. doi: 10.1186/s13104-023-06276-4 (PMC9885655; doi:10.1186/s13104-023-06276-4)
Supplement: Supplementary file 1 — Additional file1. Questionnaire. [file 13104_2023_6276_MOESM1_ESM.docx]

**QUESTIONNAIRE**

**LBP**

**Q Do you have low back pain? (LBP [yes, no])?**

□ No □ Yes

**Physical Features and Demographics**

**Q Which age group do you belong to?**

□ 20–29 □ 30–39 □ 40–49 □ 50–59

**Q Where do you live? (Present residence)**

**Q Where were you born? (Place of birth)**

□ North area □ East area □ West area □ South area

**Q Your occupation? Please choose from the following groups. (Occupation)**

□ Manager (manager/specialist/other) □ Full time □ Part time □ No job

**Q Number of breaths per minute.**

□ <15 □ ≥15.0

**Q Your height (in cm) and weight (in kg)**

Height xxx cm

Weight xx.x kg

Based on the above answers, we calculated BMI and categorized the subjects into the following groups. [BMI ＝ weight (kg) ÷ height (m)^２^]

□ <18.5 □ 18.5–24.9 □ ≥25.0

**Body temperature (BT)** hand and foot

Please use a contact-free thermometer (infrared thermometer) and measure BT in the axilla and on the forehead, hand, and foot


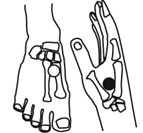


**in the axilla** xx.x °C

**on the forehead** xx.x °C

**on the hand** xx.x °C

**on the foot** xx.x °C

**Lifestyle**

**Q Present room temperature (RT ℃)**

□ <18°C □ ≥18°C

**Q Which type of heater do you use?**

□Whole room □Local □Do not have a heater

**Q How do you use the heater during the night (while you sleep)?**

□ Whole duration of sleeping

□ Until waking up

□ Only until you are about to fall asleep

□ Before waking up & until falling asleep (using a timer switch)

□ No heater used while sleeping

□ No heater used at all

**Q Your winter wear is...**

□ The same clothes that I wear throughout the year □ Warmer clothes

**Q Do you like air conditioner?**

□ Dislike □ N/A □ Like

**Q Do you currently have a history of anemia?**

□ Undergoing anemia treatment

□ Had anemia, already treated

□ Have anemia but did not undergo treatment

□ Have a disease other than anemia

□ Have not seen a doctor

**Q How often do you use antiphlogistic and analgesic agents?**

□ 2/week□ 1/week □ 3/month □ Sometimes □No history of use

**Q If you use antiphlogistic and analgesic agents, how do you obtain it?**

**(Do not answer if you have chosen** “no” **above)**

□ Doctor □ Drug store/internet □ Friend/family

**Q If you use antiphlogistic and analgesic agents, what is the reason for using them?**

**(Do not answer if you have chosen** “no” **above)**

□ During menstruation □ Back/knee pain □ Skin disorder/fever

**Q Dietary limitation**

□ Ongoing □ Sometimes □ No limitations

**Q Menses**

□ Irregular □ Painful □ No problem □ Absence of menstruation

**Q Did you catch a cold recently?**

□ No history of cold □ Yes, before last October (September yes) □ Yes, after last October

**Q The frequency of smoking cigarettes (history of smoking)**

□ No history □ Sometimes □ Very often □ Smoked/not now

**Q Do you do exercise?**

□ Yes □ No

**Q Type of exercise**

□ Anaerobic, such as sprinting □ Aerobic, such as yoga □ N/A

**Q How long do you sit in one place continuously? (Stationary hours)**

□ <1 □ 1–2 □ 2–4 □ ≥4

**Q How long do you sleep (hours)?**

□ <6 □ 6–7 □ 7–8 □ ≥8

**Q When do you go to bed? (Bedtime)**

□ Before 22:00 □ 22:00–00:00 □ 00:00–02:00 □ After 02:00

**Q How often do you go to bathroom during the sleeping hours at night? (Night bathroom)**

□ 0 □ 1 □ 2 □ ≥3

**Q Please select your bathing style?**

**□** Shower only **□** Bath (<10 min) **□** Bath (≥10 min)

**Q Do you use sauna?**

□ Yes □ No

**Q Do you have *hie*? (chilly-sensation)**

□ Yes □ No

**Diet**

**Q Do you have any tastes and distastes for specific foods? (Likes and dislikes)**

□ Yes □ N/A □ No

**Q How often do you consume cold foods?**

□ Frequently, □ N/A □ No

**Q How often do you consume fish?**

□ Frequently, □ N/A □ No

**Q How often do you consume beans?**

□ Frequently, □ N/A □ No

**Q How often do you consume fermented foods?**

□ Frequently, □ N/A □ No

**Q How often do you consume richly flavored foods?**

□ Frequently, □ N/A □ No

**Mental Status**

**Q Do you feel happy?**

**Q Do you feel angry?**

**Q Do you feel sad?**

**Q Do you feel depressed?**

**Q Do you feel inferior?**

**Q Do you feel like your health is deteriorating each day?**

**Q Do you feel exhausted?**

**Q Do you feel unsuccessful?**

□ Yes □N/A □ No
